# Plasticity in Caste-Fate Determination During the Adult Stage in Temperate *Polistes* Wasps

**DOI:** 10.3390/insects16030326

**Published:** 2025-03-19

**Authors:** Hideto Yoshimura, Ken Sasaki

**Affiliations:** 1 Division of Crop Rotation Research for Lowland Farming, Tohoku Agricultural Research Center, National Agriculture and Food Research Organization (NARO), Morioka 020-0198, Iwate, Japan; yoshimurah754@naro.affrc.go.jp; 2 Graduate School of Agriculture, Tamagawa University, Machida 194-8610, Tokyo, Japan

**Keywords:** biogenic amine, brain, caste plasticity, dopamine, eusociality, Hymenoptera, photoperiod, *Polistes*, reproduction, social insect

## Abstract

The reproductive division of labor is a fundamental characteristic of eusociality; thus, understanding the caste determination system underlying the reproductive division of labor would shed more light on the evolution of eusociality. In this review, we summarize the factors associated with caste determination in temperate *Polistes* paper wasps and focus on life histories associated with the loss or maintenance of caste plasticity during the adult stage among eusocial Hymenoptera. In many species of eusocial Hymenoptera, caste trajectories are differentiated by nutrition during the larval stages, indicating that caste plasticity is either absent or has not yet been confirmed. However, in temperate *Polistes* wasps, nutrition during the larval stage only causes biases in caste trajectory, with castes ultimately determined by environmental factors, such as day length and temperature, and colony conditions during the adult stage, indicating high caste plasticity during this stage. Therefore, morphological dimorphism and physiological differences between castes, such as in dopamine levels, have not been found in temperate *Polistes* wasps at emergence. This plasticity in temperate paper wasps could reflect the fact that females destined to be workers also have a chance to mate with males (especially early males) after emergence, leaving the possibility that they can produce daughters in the emerging year.

## Simple Summary

In this review, we summarize caste determination systems in eusocial insects, with a focus on temperate Polistes paper wasps. We describe the plasticity of caste determination, a foundational characteristic of eusociality, during the adult stage. Studies associated with caste determination have advanced in honey bees and are progressing in paper wasps. Honey bees have morphological and physiological caste differences at emergence as a result of different nutrition provision during the larval stage. By contrast, castes in temperate Polistes wasps are ultimately determined by external factors during the adult stage, with preimaginal caste biases during the larval stage; therefore, paper wasps show a high degree of plasticity of caste determination during the adult stage. Thus, studies that explore the caste determination system in temperate Polistes wasps will contribute to understanding how eusociality evolves. We also consider the factors that led to the loss or maintenance of plasticity, based on differences in life history across species, and provide insight into the evolution of eusociality in the Hymenoptera.

## 1. Introduction

The reproductive division of labor is a foundational characteristic of the eusociality of Hymenoptera [1,2]. Adult females differentiate into either queens specialized for reproduction or infertile workers engaged in labor other than egg-laying (sometimes as helpers and soldiers, depending on the type of labor). Among eusocial species, the level of social development varies depending on the degree of morphological and/or physiological differentiation between castes and the degree of behavioral specialization for the task [2,3,4,5]. The most derived level of sociality occurs in insects that are advanced eusocial, characterized by morphological dimorphism between castes, a distinct division of labor regarding reproduction and swarm nest founding [5]. A level of sociality with characteristics similar to advanced eusocial, but where nests are founded by a single female, is highly eusocial. In both advanced and highly eusocial species, females are thought to have evolved (or degenerated) the morphology required for each type of labor, such as body size, reproductive organs (number of ovarioles and developmental status of the spermatheca), exocrine glands, and brain and nervous systems [5,6,7,8,9,10,11]. By contrast, groups that lack morphological and physiological dimorphism between castes are primitively eusocial [5]. Here, differences in body size between queens and workers are continuous and sometimes caste switching can occur.

Eusocial Hymenoptera are represented by three main groups: wasps (Vespidae), bees (Apidae + Halictidae), and ants (Formicidae), and eusociality has evolved independently in each group [12,13]. This means that even social structures defined as eusocial among the Hymenoptera might differ in terms of their developmental mechanisms and the factors driving their evolution, influenced by regional and climatic differences. In addition, these groups have adapted to a range of environments, from tropical to subarctic climates [14,15,16,17]. In their expansion into temperate and subarctic regions, seasonal adaptation is important for survival, that is, they must survive winters that are unsuitable for reproduction and development [18,19]. Overwintering systems in eusocial groups in temperate zones can be divided into two types. First, both queens and workers overwinter but do not diapause (perennial species): the overwintering adults are considered to be less active than during the warm season, but begin to act immediately upon warming. Such overwintering systems are only found in advanced and highly eusocial species, represented by honey bees and ants. Second, only reproductive castes (specifically gynes, which are queen candidates) overwinter (annual species). Such overwintering systems are found in both primitively and highly eusocial species, represented by *Polistes* paper wasps, bumble bees, hornets, and yellow jackets. The gynes mate with males during the autumn, overwinter, and then initiate the founding of a nest the following spring, whereas workers cannot survive the winter. Therefore, it is possible to determine the caste by whether the female prepares for overwintering. Gynes do not develop mature eggs during the year of emergence and instead accumulate high levels of lipids [20,21,22,23]. Thus, a mechanism for sensing the seasons might be incorporated into the caste determination system in species with an annual colony cycle [24].

Caste is determined before and/or after emergence. In species with morphological and physiological dimorphism between castes, castes begin to differentiate during the larval stage and determine their morphology before emergence. This means that there is a lack of plasticity in caste determination during the adult stage. In advanced and highly eusocial Hymenoptera, caste differentiation is promoted by food quality (e.g., royal jelly) and quantity during the larval stage, and caste is already determined at emergence [2,3,10,25,26]. However, in other Hymenoptera, especially those with an annual colony cycle, such as *Polistes* wasps and bumble bees, the mechanism of caste differentiation or determination has not yet been fully elucidated.

## 2. Caste Determination System in Temperate *Polistes* Wasps

### 2.1. Preimaginal Caste-Fate Biases

In temperate *Polistes* wasps, caste-fate bias is considered to occur during immature stages (larval and pupal stages), with caste being determined at the adult stage [27,28,29,30,31,32]. Factors causing preimaginal biases include food quality/quantity, vibratory stimuli, and day length [21,30,32,33,34,35,36,37]. Food quantity during the larval stage causes differences in body sizes [33], whereby individuals with a restricted food supply (protein source) have smaller bodies [38]. Likewise, food quality during the larval stage also induces physiological differences at emergence, with individuals that consume more carbohydrates during the larval stage having a higher lipid content at emergence [35,39]. This nutrition-dependent caste determination (bias) has also been reported in highly eusocial vespid wasps [40,41], which share an origin of eusociality with Polistinae [13]. There are morphological differences between worker and gyne castes in several species of vespid wasps, considered to be affected by the amount of food after the third instar of the larval phase [42,43] (noting that some vespid wasps have a lack of morphological differences between castes, such as *Vespa ducalis*). Therefore, the nutrition-dependent caste-fate biases observed in primitive eusocial wasps might be a fundamental developmental process across eusocial wasp species and an ancestral trait of caste determination in eusocial Hymenoptera.

Vibratory stimuli are an external factor that characterizes the common ancestor of the Polistinae and Vespidae [34]. Antennal drumming behavior by adults affects the subsequent caste trajectories of larval *Polistes fuscatus*, with larvae that experience high frequencies of antennal drumming developing into individuals with a worker-type physiology at emergence [44,45]. In addition, recent work showed that the combination of nutrition and vibrational stimuli affects metabolic systems and diapause-related gene expression through different molecular control mechanisms [36]. Finally, photoperiod is also related to caste-fate biases in temperate paper wasps, whereby individuals that experience increasing light periods from the pupal to adult stage contain more developed eggs at 2 weeks after emergence (i.e., reproductive workers) [32,37]. However, it has not yet been possible to separate the effects of photoperiod and body size (perhaps indirectly reflecting larval-stage food availability), meaning that further experimental manipulation, such as combining feed limitation and day length, would clarify the effects of day length during the immature stage.

### 2.2. Imaginal Caste Determination Factors

Caste is ultimately determined by factors during the adult stage in temperate *Polistes* paper wasps [31,32,37,46,47,48]. Emerged females are divided into two types: nondiapause (mainly workers) and diapause (gynes) females. In nondiapause females, newly emerged adults can either stay or leave the natal nest. This determination is based mainly on colony status, such as the presence of queens and/or immature individuals and colony size [29,46,47,48]. The early stage of colony activity is also at high risk of disruption by predators such as birds, mammals, arthropods, and mollusks [42,49,50,51], which might also be a factor in leaving the natal nest. Nondiapause females that stay in the natal nest are engaged in internal (e.g., brood care) and/or external roles (e.g., foraging) as workers and some become reproductive workers. Within workers, there is a dominance–subordinate hierarchy in which individuals are ranked according to their aggressive behavior, with dominant individuals being more likely to be reproductive workers (generally, they oviposit unfertilized eggs and produce males) (e.g., [52,53,54,55,56]). However, this worker reproduction is generally suppressed by queen aggression and/or queen-/worker-policing behaviors (e.g., [56,57]). When the queen disappears, the most dominant female mates and becomes the successive queen [58]. In addition, in *Polistes jokahamae*, which has a dominance hierarchy based on intergroup conflict, abdominal rubbing behavior is also observed by the queen and dominant egg-laying workers [55] (note that only the behavior of reproductive workers in the queenright colony was observed in this study). This abdominal rubbing-like behavior has been reported in the tropical paper wasp *Ropalidia marginata*, in which it is thought to apply queen pheromone [59]. Therefore, it is possible that the successive queen also controls the reproductive physiology of her nestmates (i.e., fixes them to the worker caste) through pheromones.

Next, females leaving the natal nest can become either mid-season foundresses or drifters [31,33]. Mid-season foundresses found a nest by themselves and produce the next generation. In some *Polistes* species, males are produced at the same time as the first brood (often called early males). Therefore, workers have an opportunity to mate with early males and can lay both haploid and diploid eggs [54,60,61,62,63,64]. By contrast, drifters enter other colonies [65,66] and produce males [67]. Such individuals might appear primarily under long daylight (day length close to the summer solstice) because females reared individually (i.e., leaving the nest) are more likely to develop ovaries [32,37].

Finally, the decision to become a diapausing or nondiapausing individual is determined mainly by day length [32,37,68]. When adult females experience short days during the adult stage under isolated conditions, they do not develop ovaries and instead accumulate high levels of lipids in the abdomen. Thus, the day length during the adult stage is a cue to determine the time remaining until overwintering and influences caste determination. However, the photoperiod-related caste determination mechanisms might not be consistent among temperate species. The site of origin of paper wasps is considered to be in the Old World tropics [16], suggesting that temperate adaptation has likely occurred multiple times in temperate Polistinae wasps and that their photoperiodic responsiveness and intensity might also differ.

## 3. Comparison of Caste Determination Systems Between *Polistes* Wasps and Other Eusocial Hymenoptera

Eusociality in Hymenoptera has evolved convergently across three major groups (wasps, bees, and ants). Eusocial species in each group have different levels of sociality and are adapted to temperate climates. Therefore, comparison of caste determination systems between species within a group or between groups may help understand the plasticity in caste-fate determination and the evolutionary process of caste differentiation.

Based on the classification of types of social behavior (or levels of sociality) by Jandt and Toth (2015) [5], many species of temperate *Polistes* wasps are classified as primitively eusocial with no morphological differences between castes, and workers have the potential to mate and become queens, whereas temperate hornets and yellow jackets (Vespinae) are highly eusocial with different morphology. Ants are classified as highly and advanced eusocial, although morphological caste differences have been secondarily lost in some species [2]. In temperate eusocial bees, honey bees are a perennial species and classified as advanced eusocial with swarm founding. In highly and advanced eusocial species, castes are fixed at the adult stage with external morphological differentiation. Therefore, there is no plasticity in caste-fate determination during the adult stage as seen in temperate *Polistes* species.

Bumble bees are an annual species in temperate regions and reproduce a new nest by independent founding [69]. The life history of temperate bumble bees is similar to that of temperate *Polistes* wasps as discussed above. Workers in several species of *Bombus* have the potential to mate, lay fertilized eggs, and found the nest under experimental conditions [70]. In some species of bumble bees, body size overlaps between castes [3], but in the other species, body size is significantly larger in gynes than in workers, as is the size of fat body cells [71]. The larger body size in gynes might result from the storage of fat in bodies for hibernation, and the storage at 10 days after emergence is equal to that of the overwintering queen [71]. Although some temperate *Polistes* wasps respond to seasonal signals, including day length, to determine the caste fate [37,68], there are no reports of such caste determination during the adult stage in bumble bees. These bees build their nests underground, where they receive less sunlight and experience a more stable temperature compared with the nests of *Polistes* wasps in an open space without an envelope.

Molecular mechanisms of preimaginal caste determination in *Polistes* species have been studied via gene expression analyses [28,72]. Comparison of gene expression between worker-destined and gyne-destined larvae revealed that genes involved in insulin-like peptide signaling for nutrition sensing, hexamerin signaling for lipid storage, juvenile hormone binding, and oxidation-reduction activity are more highly expressed in gyne-destined larvae than worker-destined larvae, suggesting that they are candidate pathways for caste determination and/or diapause in *Polistes metricus* [28,72]. Hexameric storage proteins are also more highly expressed in gyne-destined larvae than worker-destined larvae [72,73]. These candidate pathways contain both caste determination and diapause responses, and, therefore, not all pathways may be shared with preimaginal caste determination in other eusocial Hymenoptera. In hornets and yellow jackets, the molecular mechanisms of preimaginal caste determination are still unclear. To compare eusocial wasp species with different social levels, it is necessary to elucidate the mechanisms of preimaginal caste determination in Vespidae wasps.

Mechanisms of caste determination in the honey bee are well understood and can be used as a model system in eusocial Hymenoptera for comparison to those in *Polistes* species. However, the honey bee does not diapause and, therefore, may not express diapause-related genes in queen-destined larvae. Caste determination in the honey bee is based on nutrition, especially sugar and protein during the larval stage. This activates insulin-like peptide [74,75,76], target of rapamycin (TOR) [76,77,78], and epidermal growth factor receptor (EGFR) signaling [26], increasing juvenile hormone titers in hemolymph during the larval stage [79,80]. However, the involvement of EGFR signaling pathways has not been confirmed by other studies [79,81,82] and remains controversial. Larvae with high juvenile hormone titers develop into queen-destined prepupae [83], which have a larger peak of ecdysteroid compared with worker-destined prepupae and proceed with queen-specific development [83].

In several species of temperate *Polistes* wasps, caste-fate is ultimately determined during the adult stage. In *P. jokahamae*, gyne-destined adult females exposed to a short-day photoperiod showed enhanced expression of genes involved in insulin-like peptide signaling, tryptophan metabolism, and nutrition, including the metabolism of sugars and lipids, and production of royal jelly proteins in the brain [84]. These results indicate that photoperiod-related caste determination in *P. jokahamae* involves gene expression pathways similar to those involved in preimaginal caste determination in other *Polistes* species and eusocial bees. By contrast, worker-destined queenless females exposed to a long-day photoperiod showed enhanced expression of *EGFR* and several genes involved in insulin degradation and oogenesis in the brain [84]. In honey bees, suppression of *EGFR* expression by RNAi inhibited ovarian development in queenless workers [85]. Thus, the gene expression patterns in the brains of long-day females might reflect involvement in ovarian activation and induction of worker traits.

Caste differences in physiological characteristics in the adult brain create caste-specific behavior. Dopamine is a neuroactive substance which modulates behavior in eusocial Hymenoptera and its function has been investigated in different castes [86,87,88,89,90]. The levels of dopamine in the brain are significantly higher in newly emerged queens than in emerged workers in honey bees [86], as is also the case for bumble bee castes [87]. These higher dopamine levels in the brains of gynes can contribute to queen-specific behavior in eusocial bees [88,89,90], which is in contrast to the similar levels of dopamine between castes in *Polistes* wasps at emergence [91]. Given that dopamine activates ovarian development in reproductive workers of *Polistes* wasps [92] and increases the brain levels in founding queens of *Polistes* wasps [91], similar levels of dopamine between castes suggest that both castes of temperate *Polistes* wasps have the plasticity to change reproductive states by an increase in dopamine levels during the adult stage. Another gene differentially expressed between castes in the adult brain in eusocial Hymenoptera is an insulin-like peptide. Expression of insulin-like peptides is higher in gynes than workers in adult brains of *Polistes* wasps [22,93], bumble bees [87,94], and ants [95]. Neurosecretory cells producing an insulin-like peptide distribute within the brain [95], but the neural functions are still unknown. In *Drosophila* females, insulin-like peptide signaling is upstream of juvenile hormone and dopamine production [96]. This suggests that the high expression of the insulin-like peptide receptor gene in gyne-destined adult females [84] create the gyne-specific brain physiology with higher dopamine levels.

## 4. Conclusions and Future Directions

**Figure 1:**
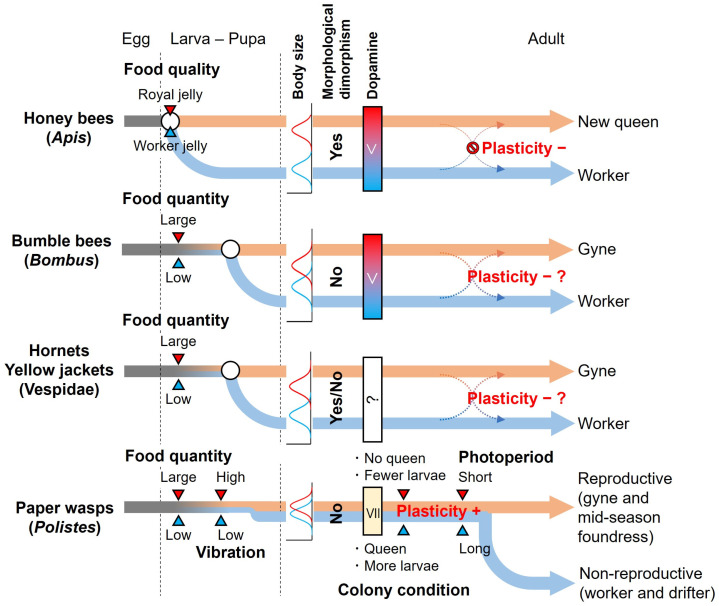
Timing of caste determination and plasticity of caste determination during adult stage in representative temperate eusocial bees and wasps. White circles indicate the timing of caste determination. In the body size column, blue and red indicate the histogram of the body size of worker (-like) and gyne (-like) individuals in the adult stage, respectively. The body sizes of honey bees and many Vespidae are bimodal distribution, while those of bumble bees and *Polistes* paper wasps are overlapping or continuous and indistinct. In addition, honey bees and some Vespidae show morphological dimorphism between castes, but bumble bees and *Polistes* paper wasps do not. In honey bees and bumble bees, there is a caste difference in the amount of dopamine of newly emerged females: the reproductive caste has higher levels of dopamine in the brain than the non-reproductive caste. On the other hand, caste differences in the amounts of dopamine of newly emerged females are much smaller in paper wasps. In adult stage plasticity, “−” and “+” indicate the group without and with plasticity of caste determination during the adult stage, respectively. In bumble bees and Vespidae wasps, no clear answer on plasticity has been obtained. The workers of these groups have not shown evidence of mating with males in the field, but they do not lose spermatheca as known in honey bees, meaning that workers have the possibility of becoming a reproductive caste. Hence, the plasticity of caste determination during the adult stage in bumble bees and Vespidae wasps is “−?”.

The plasticity of caste determination during the adult stage differs significantly between *Polistes* paper wasps and other eusocial Hymenoptera, such as bumble bees: temperate *Polistes* paper wasps have a high plasticity of caste determination during the adult stage, whereas bumble bees have caste determination during the larval stage and low plasticity during the adult stage (Figure 1). Why is there such a large difference in the plasticity of caste determination in the adult stage among primitive eusocial groups? Considering the evolution of social structure from solitary to eusocial, plasticity originally existed in all individuals, and then, during the evolution of social structure, some groups or species lost plasticity in the adult stage [97,98]. Here, we consider the factors that led to the loss or maintenance of plasticity, based on the differences in their life histories.

First, in many temperate eusocial wasps and bees, larval food quantity and/or quality is an important factor in bias toward caste differentiation or caste decision [26,33,35,36,38,39,42,43]. However, food sources differ significantly between wasps and bees. Wasps feed their larvae on both animal (protein) and plant (nectar) food resources with progressive provisioning, whereas bees feed on only plant food resources (pollen and nectar). In addition, honey bees and some bumble bee species store larval food in the cell (i.e., mass provisioning). Considering the stability of the food supply for larvae, mass provisioning is more stable than progressive provisioning. Hence, the larvae of the species with mass provisioning might be able to determine their caste at immature stages because they can fully develop without being affected by unstable and uncertain environmental conditions, such as a lack of food. However, there are counterarguments to this hypothesis. In *Polistes* paper wasps, larval cannibalism has been observed when the colony experiences restricted food availability [99,100]; thus, the larvae can serve as an animal resource (i.e., protein storage) in situations of food deprivation. Such protein storage may be a means of temporarily tolerating the unstable environment. Moreover, temperate Vespidae wasps also have distinct morphological differences between castes, even though they display the same progressive provisioning as the *Polistes* paper wasps. These differences cannot be explained by food quantity. Recently, LeBoeuf et al. (2016) [101] revealed that many growth-related proteins and juvenile hormones are included in the trophallaxis fluid exchanged between adults and larvae. Research on trophallaxis in Vespinae wasps has focused on larvae to adults [102], and future work to determine the components of trophallaxis fluid in adults to larvae may provide clues to understand morphological caste differences.

Second, the nest structure differs greatly between temperate *Polistes* wasps and other eusocial Hymenoptera: *Polistes* paper wasps found a nest in an open space without an envelope. However, hornets and yellow jackets typically build a nest covered with an envelope in an open space and/or in closed spaces, such as underground or in tree hollows [41], as do temperate honey bees and bumble bees, although bee hives are not covered with an envelope. Nests built in enclosed spaces and covered with an envelope could protect larvae from harsh weather and maintain a consistent temperature [103,104,105], resulting in a stable developmental period during the immature stage.

Finally, several options to increase direct fitness after emergence may contribute to maintaining the plasticity of caste-fate determination during the adult stage. In particular, whether they have an opportunity to mate and produce both females (fertilized eggs) and males (unfertilized eggs) in the emerging year would be a significant point. Some temperate *Polistes* paper wasps produce early males, as described in Section 2.2. These early males mate with early-emerged females (usually considered to be workers) and mated female individuals can produce both males and females [54,61,62,63,64]. Early males have also been identified in hornets [106], but it is not known whether they mate with early-emerged females. However, early male production is rare in bumble bees; therefore, early-emerged females are unlikely to produce female offspring. Future comparisons of the percentage of early males in the field or the degree of female production by early females and the degree of plasticity of caste determination during the adult stage among groups of eusocial species and among species within each eusocial group could provide clues to understanding why there is such a large difference in the plasticity of caste determination in the adult stage among primitive eusocial insects.

In conclusion, we introduced preimaginal and imaginal caste determination systems in eusocial Hymenoptera and compared caste determination systems between *Polistes* species and other temperate eusocial species. We have discussed three possible factors that maintain the plasticity of caste determination during the adult stage in temperate *Polistes* wasps from the perspective of life history by comparing with other eusocial Hymenoptera groups: (1) the stability of food availability during the larval stage, (2) nest structure, and (3) an opportunity to mate and produce fertilized eggs in the emerging year. However, none of the three factors in this review is sufficient to completely explain plasticity based on current research findings. Therefore, a first step for a future study would be to determine the degree of variation in the strength of the plasticity during the adult stage among temperate *Polistes* species. In addition, it would be necessary to steadily clarify the pieces of life history and caste determinants (e.g., function of the early male in bumble bees and Vespidae wasps, and physiology of reproductive regulation in Vespidae wasps).

## Data Availability

No new data were created or analyzed in this study.
